# Biomedical Applications and Bioavailability of Curcumin—An Updated Overview

**DOI:** 10.3390/pharmaceutics13122102

**Published:** 2021-12-07

**Authors:** Soo-In Sohn, Arumugam Priya, Boopathi Balasubramaniam, Pandiyan Muthuramalingam, Chandran Sivasankar, Anthonymuthu Selvaraj, Alaguvel Valliammai, Ravi Jothi, Subramani Pandian

**Affiliations:** 1 Department of Agricultural Biotechnology, National Institute of Agricultural Sciences, Rural Development Administration, Jeonju 54874, Korea; 2 Department of Biotechnology, Alagappa University, Karaikudi 630003, India; priya6bt@gmail.com (A.P.); pandianmuthuramalingam@gmail.com (P.M.); navinabiotech@gmail.com (R.J.); 3 Department of Molecular Biology, University of Wyoming, Laramire, WY 82071, USA; bbalasub@uwyo.edu; 4 Department of Biotechnology, Sri Shakthi Institute of Engineering and Technology, Coimbatore 641062, India; 5 Department of Food Science and Technology, Pondicherry University, Pondicherry 605014, India; biosivam@gmail.com; 6 Department of Physiology and Biophysics, University of California, Irvine, CA 92697, USA; aselvar3@uci.edu; 7 Department of Environmental Hydrology and Microbiology, Ben-Gurion University of the Negev, Beersheba 84990, Israel; alaguvel@post.bgu.ac.il

**Keywords:** curcumin, bioavailability, antibiotics, anticancer agents, nanomedicine, pharmaceutical formulations

## Abstract

Curcumin, a yellow-colored molecule derived from the rhizome of *Curcuma longa*, has been identified as the bioactive compound responsible for numerous pharmacological activities of turmeric, including anticancer, antimicrobial, anti-inflammatory, antioxidant, antidiabetic, etc. Nevertheless, the clinical application of curcumin is inadequate due to its low solubility, poor absorption, rapid metabolism and elimination. Advancements in recent research have shown several components and techniques to increase the bioavailability of curcumin. Combining with adjuvants, encapsulating in carriers and formulating in nanoforms, in combination with other bioactive agents, synthetic derivatives and structural analogs of curcumin, have shown increased efficiency and bioavailability, thereby augmenting the range of applications of curcumin. The scope for incorporating biotechnology and nanotechnology in amending the current drawbacks would help in expanding the biomedical applications and clinical efficacy of curcumin. Therefore, in this review, we provide a comprehensive overview of the plethora of therapeutic potentials of curcumin, their drawbacks in efficient clinical applications and the recent advancements in improving curcumin’s bioavailability for effective use in various biomedical applications.

## 1. Introduction

Bioactive compounds with therapeutic value are found in a huge spectrum of natural resources. Among them, phytochemicals from plants have gained greater interest for mankind ever since ancient times. Various research groups have analyzed diverse groups of plant-based bioactive compounds to discover their pharmaceutical efficiency in treating various kinds of human illness [1]. In traditional Indian and Chinese medications, the turmeric plant, *Curcuma longa* Linn., has been widely used, owing to its therapeutic magnitude [2]. One of the major bioactive components of the rhizome extract of turmeric is curcumin, a yellow-colored hydrophobic polyphenol. Chemically, curcumin is known as 1,7-bis(4-hydroxy-3-methoxyphenyl)hepta-1,6-diene-3,5-dione or, precisely, diferuloylmethane [3]. Aroma and color are the primary characteristics of curcumin in the diet. Though no nutraceutical value was found, numerous research articles have shown the therapeutic potential of curcumin, which includes but is not limited to being antioxidant, anticarcinogenic, antimicrobial, anti-inflammatory, hypoglycemic, hepato- and neuroprotective [4,5,6], etc. Due to its broad spectrum of therapeutic efficiencies, researchers are engrossed in unearthing the molecular targets and their encoding mechanisms of action of curcumin that underlie these biological activities. The nontoxic nature of curcumin makes it an ideal therapeutic agent, yet the limited bioavailability is a major concern in clinical application [7]. Due to its poor absorption, rapid metabolism and rapid elimination, the concentration of curcumin available in plasma and target tissue becomes low. In recent times, several studies have devised methods to increase the bioavailability of curcumin. Curcumin complex with the following adjuvants, such as nanoparticles, micelles, phospholipid complexes, liposomes, etc., have been used to increase the bioavailability. For instance, piperine, a major alkaloid from black pepper, is known to enhance the bioavailability of curcumin by 2000% [5]. Advanced research on adjuvants has shown enhancements in bioavailability which, in the near future, could bring promising clinical outcomes for curcumin treatment. Encapsulation of curcumin in nanostructures has shown enhanced therapeutic efficiency by extending the half-life of the drug, enhancing the solubility and sustained release of the drug [8]. Curcumin encapsulation into carriers such as liposomes, micelles, polymeric nanoparticles, mesoporous silica nanoparticles or nanoformulations has been used for a variety of applications, such as cancer therapy [9], wound healing application [10], diabetic complications [11], antimicrobial agents [12], neuroprotection [13] and so on. This review provides a comprehensive overview of the structure and the recent advancements in the biomedical applications of curcumin, use of nanotechnology to produce nanoencapsulated curcumin, significance of curcumin bioavailability and current trends in improving bioavailability.

## 2. Biosynthesis Pathway and Structure of Curcumin

Curcumin (diferuloylmethane) (C_21_H_20_O_6_), an orange-yellow component of turmeric, is a polyphenol natural product extracted from *C. longa*. Significantly, this natural polyphenol is commonly called the “wonder drug of life” [14]. Curcumin has been used in various forms for centuries around the world for a variety of potential health benefits, as well as a food-coloring agent [15]. The chemical composition of curcumin has been well known since the eighteenth century. According to the current IUPAC rules, the name of curcumin is 1,7-bis(4-hydroxy-3-methoxyphenyl)hepta-1,6-diene-3,5-dione [3,16]. The molecular formula and weight of the curcumin are C_21_H_20_O_6_ and 368.38 g/mol and other physicochemical properties of curcumin, such as lipophilicity (ADME: cLogP, cLogS), pharmacokinetics (gastrointestinal absorption, blood–brain barrier (BBB)) and druglikeness (bioavailability), are given in Table 1 [17]. The - curcumin has three different chemical substances, such as two aromatic ring systems: o-methoxy phenolic groups, linked by a seven-carbon connector comprising an α, β-unsaturated β-diketone moiety [18]. In general, curcumin and its chemical structure make it less soluble in water at both acidic and neutral pH. On the other hand, curcumin is soluble in alkali, acetic acid, acetone, chloroform, DMSO, ethanol and ketone [19].

The turmeric rhizome contains a complex of curcuminoids, and it has various methoxy groups: bisdeshydroxybisdesmethoxycurcumin, bisdemethoxycurcumin and demethoxycurcumin, and curcumins are significantly responsible for several biological and pharmacological activities that are significant in treating many complex disorders/diseases.

**Figure 1:**
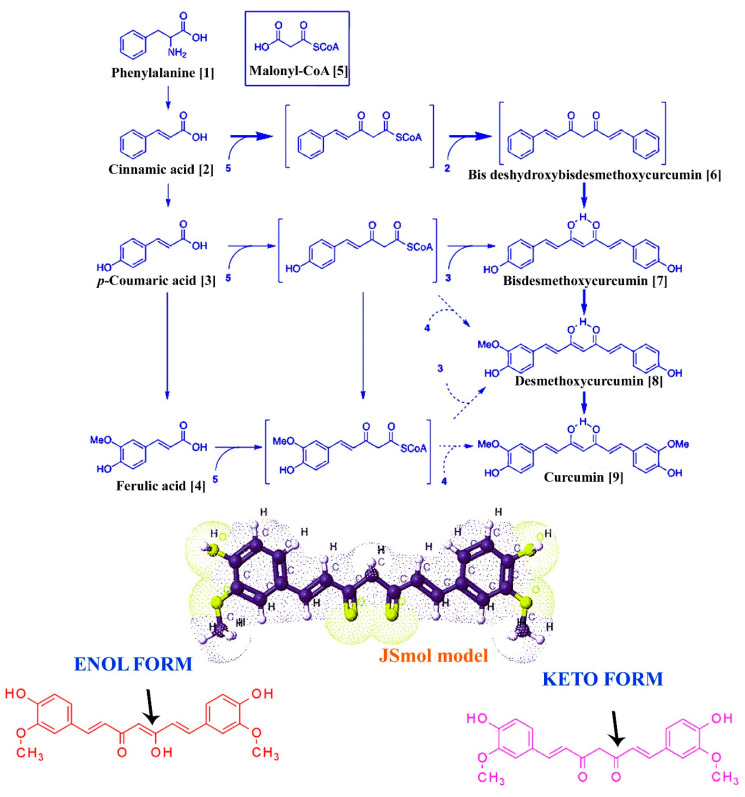
Basic structure and biosynthesis of curcumin. The figure represents the partial biosynthesis pathway of curcumin in turmeric: the JSMol model, enol and keto form of curcumin.

In particular, curcuminoids are synthesized by type III polyketide synthases (PK-S) in plants (Figure 1) and contain two phenylpropanoid molecules which are chemically derived from the aromatic amino acid, namely, phenylalanine (1), and interact by a central carbon unit originally derived from malonyl coenzyme A (malonyl-CoA (5) is also known as an extender substrate, providing sequential cyclization of the PK chain [20]). Further, cinnamic acid (2) is produced from phenylalanine (1) with the help of catalyzing enzyme phenylalanine ammonia lyase (PAL) and converted into *p*-coumaric acid (3) by cinnamate-4-hydroxylase (C4H) and 4-coumarate-CoA ligase (4CL). Then, 4CL converts the *p*-coumaric acid to coumaroyl-CoA, *p*-coumaroyl shikimate transferase (CST), caffeoyl-CoA *O*-methyltransferase (CCoAOMT) and *p*-coumaroyl 5-*O*-shikimate 3-hydroxylase (CS3=H) convert it to ferulic acid (4). Then, a combination of CoAs (i.e., coumaroyl-CoA and feruloyl-CoA) are formally converted to diketide-CoAs with the help of diketide-CoA synthase (DCS) by condensation of malonyl-CoA. At the end of the biosynthetic process, curcumin synthases (CURSs) catalyze the formation of curcuminoids via condensing the diketide-CoAs in combination with coumaroyl-CoA and feruloyl-CoA. Based on the combination, types of curcuminoids are synthesized/produced, namely, bisdeshydroxybisdesmethoxycurcumin (6), bisdemethoxycurcumin (7), demethoxycurcumin (8) and curcumin (9).

**Figure 2:**
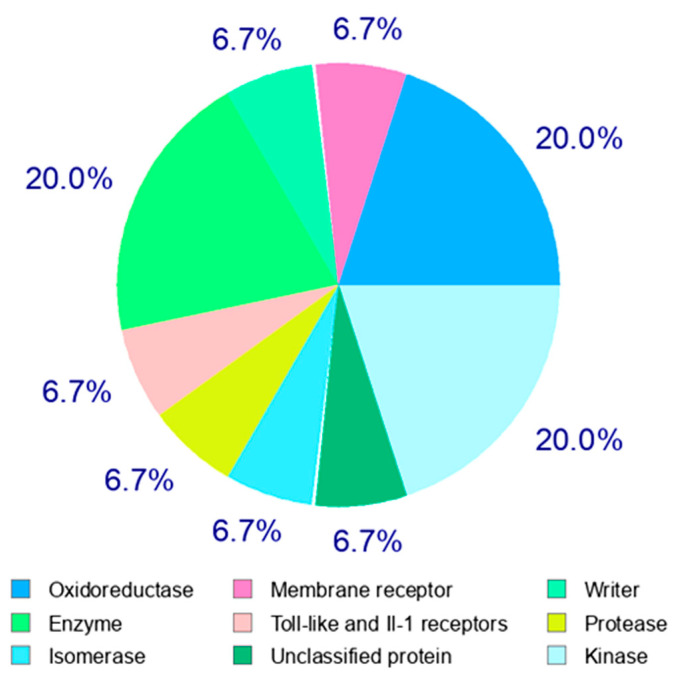
Target classes of curcumin. Among the various classes of enzymes, curcumin majorly targets oxidoreductases, kinases and several other enzymes, as stated in the image.

Curcumin has a high potential ability to bind and alter the regulation of different proteins, growth factors, transcription factors, enzymes, receptors and other regulatory genes directly/indirectly [21]. So far, several researchers have comprehensively investigated the targets of curcumin in human beings and identified the direct targets as including protein kinases/reductases, proteosomes, inflammatory molecules, carrier proteins, DNA methyltransferases and metal ions. Enzymes, transcription factors, growth factors, receptors, inflammation mediators, cell cycles and adhesion molecules are considered as indirect targets as well [15]. Curcumin is a pleiotropic molecule that can interact with a variety of molecular targets. The classes of interactions mediated by curcumin were predicted by the SwissTargetPrediction tool and are summarized in Figure 2 and Supplementary Table S1.

## 3. Biological Properties and Biomedical Applications

**Figure 3:**
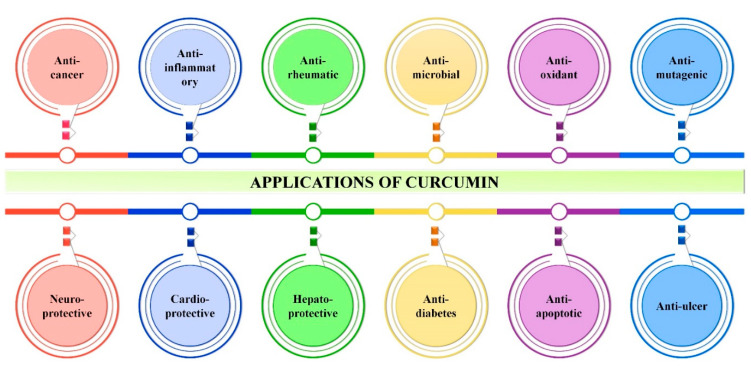
Overall applications of curcumin. Image depicts various biological applications of curcumin irrespective to its bioavailability.

The medicinal usage of curcumin was started more than 2500 years ago in Asia, especially by the native population of India. In the scientific community, the medicinal use of curcumin was first reported to be against chronic cholecystitis and allied diseases in 1937. To date, there are more than 8000 studies/articles available in the PubMed database demonstrating the various biological properties of curcumin, such as antioxidant, antibacterial, antifungal, antiviral, anti-inflammatory, anticancer, antiproliferative, proapoptotic and anti-atherosclerotic effects, and its medicinal benefits against various forms of human illnesses, including neurodegenerative diseases, arthritis, allergy, inflammatory bowel disease, nephrotoxicity, diabetes, multiple sclerosis, cardiovascular disease, lung fibrosis [3,18,22], AIDS [23] and psoriasis [24] (Figure 3). As stated, curcumin has various biological applications for which the molecular mechanisms of these properties are presented in Table 2.

### 3.1. Anticancer Effect

**Figure 4:**
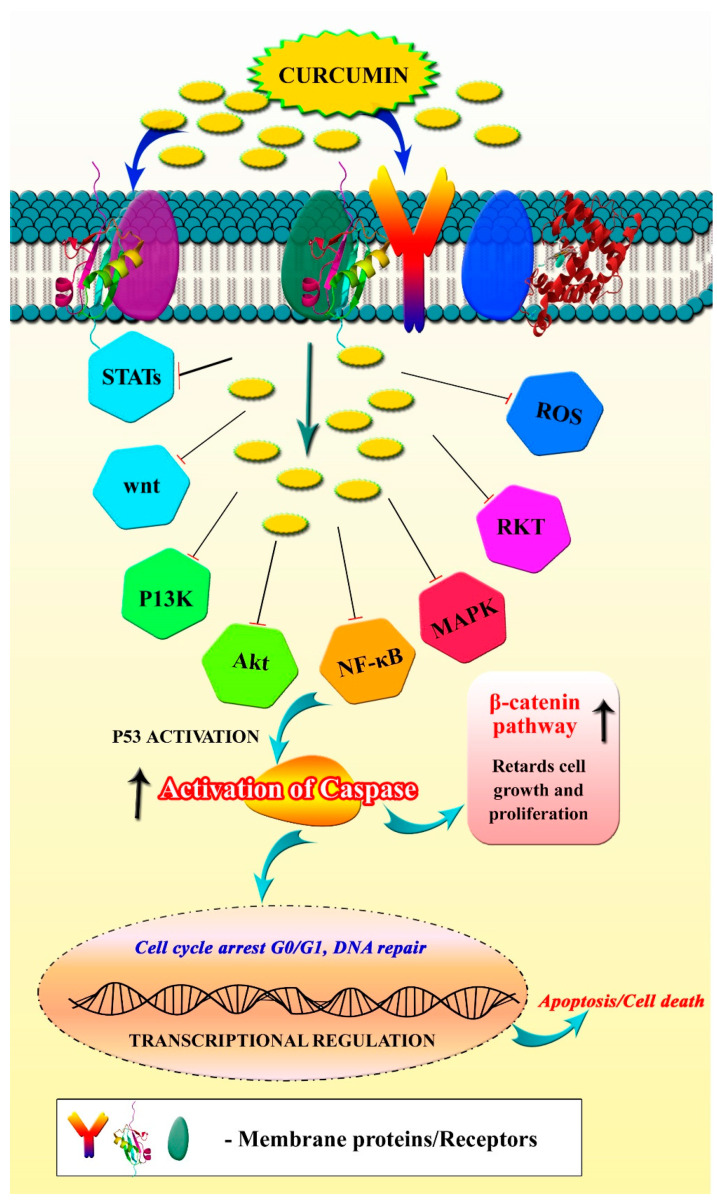
Anticancer mechanism of curcumin. Image shows the regulatory targets/pathways of curcumin in anticancer activity.

Though curcumin has been reported for its inhibitory efficacy against a variety of diseases, its anticancer effect is capturing the attention of cancer researchers. A plethora of cancer prevention research studies have demonstrated the efficacy of curcumin in both preventing and treating cancer, as well as described its various mechanisms of action [43]. According to several research studies, curcumin hinders the proliferation of cancer cells by the following mechanisms: (i) downregulation of the NF-B/STAT3 signaling pathway [44], (ii) inhibition of different regulatory protein kinases [45], (iii) regulation of apoptosis and other cancer-related pathways [46], (iv) promoting cell cycle arrest [47], (v) suppression of metastasis factors and (vi) promoting ROS-mediated cancer cell death. The inhibitory effect and mode of action of curcumin on cancer cells are illustrated in Figure 4.

#### 3.1.1. Downregulation of NF-κB/STAT3 Signaling Pathways

The transcriptional factor NF-κB is responsible for the regulation of certain genes that promote cancer cell proliferation, including cyclin D1, and the cyclin-dependent kinase inhibitors p21 and MMP-1 and MMP-3 genes [48]. The continuous activation of NF-κB can promote cancer cell proliferation and development via suppressing apoptosis. A study by Liu et al. [49] demonstrated that the anticancer activity of curcumin against breast cancer is mainly via downregulating the NF-κB-inducing genes. It was believed that downregulation of NF-κB genes by curcumin could plausibly suppress other downstream genes that may be responsible for cancer cell proliferation.

Similarly, curcumin was also found to inhibit the pathways that are mediated by another transcriptional factor, namely, signal transducer and activator of transcription 3 (STAT3), which is solely responsible for cancer cell survival, angiogenesis and metastasis. Curcumin demonstrated antivasculogenic mimicry (VM) activity in hepatocellular carcinoma cells by inhibiting/suppressing the STAT3 and AKT signaling pathways [50]. In addition, curcumin has been shown to hamper the STAT3 phosphorylation mediated by IL-6 in myeloma cells [31,51].

#### 3.1.2. Inhibition of Protein Kinases

Kinases are a group of enzymes whose function is to transfer the phosphate group to the target/substrate protein via the process called “phosphorylation”. Though phosphorylation is a reversible process, activated kinases can aid the cancer cell by inducing angiogenesis, metastasis and cell multiplication and also thwart the event of apoptosis. Curcumin has been shown to suppress several cancer-related kinases, including AMP-kinase, S-phase kinase-associated protein 2 (Skp2) pathway, mitogen-activated protein kinase (MAPK) (p38, ERK) and AKT/protein kinase B (PKB).

A study by Chen et al. [52] revealed that curcumin has the VM effect by downregulating the expression of the erythropoietin-producing hepatocellular receptor tyrosine kinase class A2 (EphA2)/PI3K/MMP signaling pathway in a murine choroidal melanoma model. Another research study revealed the anticancer mode of action of curcumin against human epithelial cancer cells (A431) and unveiled the fact that curcumin manifestation could inhibit the EGFR tyrosine kinase by promoting autophosphorylation [53]. Previous reports revealed that the antiproliferative and antimetastatic effects of curcumin were mediated by inducing the phosphorylation of AMP-kinase in breast cancer cells [54].

#### 3.1.3. Regulation of Apoptosis

Apoptosis is the fundamental, natural mechanism in which unhealthy/unwanted cells undergo programmed cell death. This natural phenomenon is vital to balancing inner cell homeostasis. Any apoptosis misbehavior can result in abnormal cell growth and contribute to the progression of cancer [55]. Although curcumin has been shown to target different molecular pathways, in most of the research studies it has been demonstrated that the anticancer mechanism of curcumin is mainly via apoptosis. A myriad of research studies has reinforced the fact that curcumin can balance apoptosis either through increasing the expression of proapoptotic genes or by downregulating certain antiapoptotic genes. For instance, curcumin has been identified as possessing anticancer activity through promoting apoptosis in scleroderma lung fibroblasts [56]. Furthermore, numerous studies have shown that curcumin modulates important apoptotic proteins such as BAX, BCL-2, C-MYC, BCL-XL and p53 and, as a result, induces apoptosis in human hepatoma SMMC-7721 cells [57], mammary epithelial and breast carcinoma cell lines [58].

Furthermore, previous studies revealed the ability of curcumin to modulate various apoptotic signaling pathways that, ultimately, result in the downregulation of certain antiapoptosis genes (Bcl-2) or upregulation of proapoptosis genes (Bax, PUMA and caspase cascades) [46]. In addition, curcumin has been found to activate the JNK and ER stress apoptotic pathways, which are mainly mediated through the accumulation of reactive oxygen species (ROS) in gastric cancer cells [59]. Some more studies revealed that curcumin is able to downregulate the expression of several genes, such as protein tyrosine kinase, protein kinase C, c-myc mRNA expression and bcl-2 mRNA expression, that lead to the induction of apoptosis in cancer cells [30].

#### 3.1.4. Promoting Cell Cycle Arrest

In mammals, the cell cycle is coordinated by five distinct phases that include G1, G2 (nucleic acid and protein synthesis phase), S (DNA-replicating phase), M (mitosis phase) and G0 (resting phase) [60]. Any malfunction in the cell cycle regulatory check points can lead to tumor formation. Derangements in the regulatory control of the cell cycle can result in the formation of tumor cells in which growth and proliferation proceed unchecked. According to Wu et al. [61], curcumin induced G2/M cell cycle arrest by inhibiting protein kinase 1 (DAPK1), which resulted in the inhibition of STAT3 and NF-B, which further activated caspase-3. Research studies also documented that the modulation of the miR-21 gene by curcumin could cause G2/M phase cell cycle arrest in HCT 116 colorectal cancer cells [62]. It has also been observed that the downregulation of certain cancer-associated genes such as tubulin and p53 by curcumin could also cause cell cycle arrest at the G2/M phase in the treatment of colon cancer [63]. In addition, curcumin similarly acted on Aurora-A SiRNA (small interfering RNA), leading to monopolar spindle formation, S and G2/M arrest and cell division reduction in MCF-7 cells.

#### 3.1.5. Suppression of Metastasis Factors

Metastasis is a process whereby cancer cells can spread from the original site to new potential locations, mostly carried out through the circulation of the bloodstream or lymph system [64]. Curcumin has the potential to inhibit cancer cell metastasis through the regulation of various cancer-associated factors, such as inflammatory cytokines, growth factors and growth factors’ receptors, enzymes, adhesion molecules, apoptosis and cell-cycle-related proteins [65]. In particular, curcumin has been reported to inhibit metastasis by blocking the activation of STAT3 (which is associated with cancer cell metastasis). Curcumin hinders the signal transduction needed for the phosphorylation and activation of STAT3 via binding to the Janus-activated kinase (JAK) activation loop [66]. Curcumin also blocks metastasis through the inhibition of focal adhesion kinase (FAK) activity. A study by Chen et al. [52] demonstrated the metastasis inhibitory efficacy of curcumin in both in vitro and in vivo conditions. From the study, they conclude that exposure to curcumin could potentially downregulate the expression of one of the tumor adhesive molecules (CD24), resulting in the suppression of FAK phosphorylation [52].

#### 3.1.6. Promoting ROS-Mediated Cancer Cell Death

In cancer cells, ROS showcased double-sided effects; it can trigger or suppress the cancer cell expression. Several studies have shown that the anticancer mechanism of curcumin is either through scavenging or induction of ROS generation. Khan et al. [31] demonstrated the ROS-mediated apoptosis in T-cell lymphoma (HuT-78) cells. They suggested that rapid generation of ROS by curcumin could potentially activate the caspase activity, alter the mitochondrial membrane potential and downregulate the expression of antiapoptotic proteins c-FLIP and Bcl-xL [31,67]. Evidence suggested that ROS-mediated upregulation of DR5 and activation of TRAIL-induced apoptosis were observed in renal cancer cells and colon cancer cells that were manifested with curcumin [68,69]. In gastric tumor cells, curcumin induced the rapid ROS generation and, thereby, reduces the cellular bioenergetics, i.e., decreased mtDNA content and DNA polymerase γ (POLG), resulting in cancer cell death [70]. It has also been reported that curcumin induces ROS generation to the threshold level and suppresses the ROS multiple enzymes to inhibit the cancer cell growth in human erythroleukemic K562 cells [71].

### 3.2. Antioxidant and Radical Scavenging Properties

ROS is continuously produced by all aerobic organisms during respiration and some physiological events. If ROS is not scavenged, it could be responsible for developing more than 100 diseases. To avoid these issues, every aerobic microorganism has a specific mechanism to protect itself from ROS (antioxidant property) [72]. Numerous research studies have shown the potential antioxidant and radical scavenging properties of curcumin in both in vivo and in vitro models. Most of the studies confirm that the pharmacological/therapeutic activity of curcumin is mainly attributed to its ability to scavenge reactive free radicals. Previous studies showed the antioxidant property of curcumin and its derivatives (demethyoxycurcumin, bisdemethoxycurcumin and diacetylcurcumin) and, from the results, they concluded that curcumin protects hemoglobin oxidation through its antioxidant property in macrophages [25,26]. Another study found that curcumin can be a “superb antioxidant” agent by donating its H-bond. They also suggested using curcumin in various pharmaceutical products to inhibit lipid oxidation and to improve the medicinal quality of products [73]. Curcumin administered orally to male Wistar rats for 10 days could reduce iron-induced hepatic damage by reducing ROS-induced lipid peroxidation [74]. It was highly controversial whether the antioxidant property of curcumin was attributable to the phenolic or the central methylenic hydrogen. Many researchers have been trying to find the solution. In both acidic and neutral environments, Jovanovic et al. [75] found that the H atom was donated from the central methylenic group of curcumin. Contrary to this result, Barclay et al. suggested that the H atom in curcumin was donated from its two phenolic groups and not from enol [76]. In agreement with this point, another study claimed that the phenolic group of curcumin is mainly responsible for its free radical scavenging property [77].

### 3.3. Anti-Inflammatory Properties

Though inflammatory responses by immunological cells are considered a necessary step in protecting against infections, undesired inflammation may lead to the development of disease, e.g., rheumatoid arthritis. Curcumin has been shown to modulate the different mediators that are solely responsible for uncontrolled inflammation [4]. The most accepted anti-inflammatory response of curcumin is mediated through (1) downregulation of cyclooxygenase-2 (COX-2), lipoxygenase and inducible nitric oxide synthase (iNOS), (2) inhibition of cytokine production and (3) reduction of transcriptional factors [22]. COX-2 is an enzyme that mediates the conversion of prostaglandins (PGs) from arachidonic acid. The overexpression of PGs may induce the overgrowth of tumor cells in both human beings and experimental animals [78]. It has been reported that constant upregulation of COX could potentially be responsible for various tumor developments such as colon, rectum, breast, head and neck, lung, pancreas, stomach and prostate cancer cells [79]. Several studies have shown that curcumin possesses anti-inflammatory activity by blocking the overexpression of COX-2 in different cell lines and animal models [80]. Topical application of curcumin has been shown to reduce the expression of PGs by blocking the lipopolysaccharide-mediated COX-2 expression [36,80]. As discussed previously, the transcriptional factor NF-κB plays a vital role in cancer cell proliferation, oncogenesis and cell transformation via mediating inflammatory responses. An experimental study has shown that oxidative stress mediates NF-κB inhibition of curcumin [81]. Singh et al. [82] explained that the curcumin mediated the NF-κB activation pathway inhibition a step before I kappa B-alpha phosphorylation but after the convergence of various stimuli.

### 3.4. Antimicrobial and Antibiofilm Activities of Curcumin

**Figure 5:**
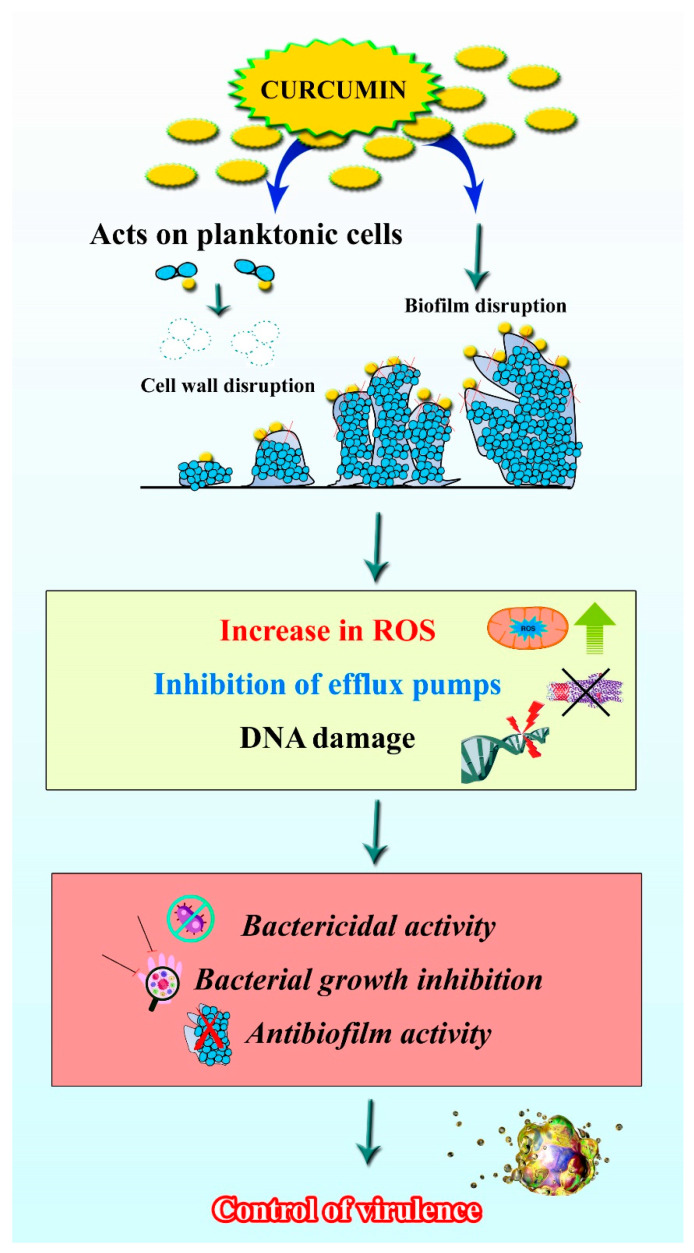
Antibiofilm activity of curcumin. Image shows that curcumin can act on the planktonic cells, as well biofilms of certain bacteria by increasing the ROS, inhibiting the efflux pumps and causing DNA damage.

The rhizome of *C. longa* has been traditionally used as an antimicrobial agent [83]. Curcumin has been used for a variety of purposes, and topical application of curcumin on the skin can help with antimicrobial care for wounds, acne, blisters, parasitic infections and other conditions [84]. For common colds, liver infections and urinary tract infections, oral administration was preferred [85]. Due to its comprehensive antimicrobial activity and innocuous nature, even at higher dosage concentrations, the structure of curcumin is being used as a prototype to design new antimicrobials and synthetic derivatives of curcumin with increased efficiency [85]. The structure of curcumin has conjugates such as two methoxylated phenols and a β-diketone in its enol form, which enables additional inter- and intra-hydrogen bonds. However, the presence of hydroxyl and methoxyl groups is considered to be accountable for antimicrobial activity [86]. The ability to inhibit the growth of various microscopic organisms has paved the way for curcumin’s numerous applications, including in the textile industry for the fabrication of antimicrobial clothes, wound dressing, photodynamic therapy in dental applications, nanoformulations for burn wounds, etc. [87,88]. In this context, curcumin targets the ROS mechanism of the microorganism, efflux pumps and causes DNA damage to control cell growth, as shown in Figure 5.

#### 3.4.1. Antimicrobial Fabric

Among the indoor microbial contaminations of several industries, the textile industry is more susceptible to the growth of pathogenic organisms. Factors such as large surface area, moisture content, etc., provide a favorable environment for the microbial colonization, which often leads to deterioration in the quality of the fabric and an unpleasant odor that affects the product as well as those who wear it [89]. To overcome these shortcomings, a variety of novel approaches have been implemented. One such is the use of natural ingredients as antimicrobial agents as a final glaze for fabrics. Curcumin is commonly used for this purpose due to its dual function, viz., natural dye and antimicrobial property. Numerous studies have reported that wool fabric treated with curcumin exhibits increased antimicrobial activity even after prolonged wash cycles [87,90]. Certain researchers have synthesized novel dyes based on curcumin. Gaffer et al. [91] synthesized curcumin containing sulphadiazine and sulphathiazole dyes for application in antimicrobial silk fabrication. Antibacterial medical textiles with curcumin were also fabricated as a curcumin-based water-dispersal polyurethane textile finish [92]. Several investigations have described the proficiency of curcumin in antimicrobial wound dressing applications as well [36,93,94,95].

#### 3.4.2. Antimicrobial Photodynamic Therapy

Photodynamic therapy (PDT) has been a useful therapeutic option to treat various solid tumors for decades with the administration of photosensitizing agents and light of specific wavelengths [96]. Due to the constant increase in antimicrobial resistance and the decline in the effectiveness of antibiotics, antimicrobial photodynamic therapy (aPDT) has been used as one of the alternative approaches to eradicate infections caused by resistant pathogens [97]. aPDT has been used as a treatment for various infections caused by bacteria, fungus and viruses [98]. Curcumin has been identified as a potential photosensitizing agent [99] since the absorption maximum of curcumin is in the blue wavelength range (420–430 nm) and the penetration depth is comparably lower than that of red light. Thus, curcumin as a photosensitizer in PDT is limited to treatment for topical infections [100]. Curcumin-mediated aPDT has been demonstrated as an effectual therapy against oral infections such as dental plaque [101], periodontitis [102] and oral candidiasis [103]. Dosage concentration, incubation time and broad-spectrum antimicrobial activity against both Gram-positive and Gram-negative bacteria, fungi, etc., enhance the efficiency of curcumin in aPDT significantly [104].

#### 3.4.3. Antimicrobial Combinations with Curcumin

Among various strategies to combat antimicrobial resistance, combinatorial drug therapy has gained more attention due to the benefits of reduced toxicity, improved efficacy and lack of antibiotic resistance with greater potential than individual drug candidates [105]. Curcumin has shown excellent synergistic activity with various antibiotics, as well as other natural bioactives. The combination of curcumin and polymyxins has demonstrated promising antimicrobial effects against multidrug-resistant pathogens. In addition to increased antimicrobial activity, curcumin–polymyxin combination therapy has reduced the polymyxin-induced nephron and neurotoxicity [106]. Another study found that the interaction of quercetin and curcumin exhibited synergistic antimicrobial activity against MRSA, as well as improved anti-inflammatory activity [107]. Several other in vitro studies have shown that various active compounds have successful antimicrobial combinatorial proficiency against a wide range of multidrug-resistant pathogens [108,109].

#### 3.4.4. Antimicrobial Activity of Curcumin in Nanoformulations

In recent decades, nanomedicine has been rising as a promising field. Nanoscale bioactive structures have shown increased physical and chemical properties and thus are utilized in numerous diagnostic and therapeutic applications [18]. Due to the hydrophobic nature of curcumin, its modification to nanoform has shown increased therapeutic efficiency, bioavailability and stability [110]. The most common forms of curcumin nanoformulations include nanoparticles, nanoencapsulation, micelles and liposomes [17]. Curcumin nanoencapsulations and nanocrystals have demonstrated antimicrobial activity against several Gram-positive and Gram-negative pathogens, including *S. aureus*, *E. coli*, *P. aeruginosa*, *L. monocytogenes*, *S. epidermidis*, etc. [111,112,113]. Similarly, the nanogel form of curcumin has been used as a topical antimicrobial for wound burns [114]. It is not only limited to antibacterial activity; it possesses a broad spectrum of antimicrobial activity when it is formulated using nanotechnology, by which it can act against fungi [115], parasites [116] and also viruses [117].

#### 3.4.5. Antibiofilm Activity

Biofilm is the most common form of microbial lifestyle, wherein organisms are associated with various kinds of surfaces. Pathogens are protected from host defense and environmental factors by encasing them within the self-produced matrix of exopolymeric substances [118]. Biofilm formation has a significant impact on the rise of antimicrobial resistance. Hence, antibiofilm molecules which can arrest or disrupt the biofilms of pathogenic organisms are part of the current trend [119]. In addition to all the therapeutic efficiencies, curcumin has been shown to possess antibiofilm activity against several organisms. Sethupathy et al. [120] displayed the efficacy of curcumin in inhibiting the virulence attributes and biofilm formation of *P. aeruginosa* and its clinical isolates without affecting the growth. The study also reported that the mechanism underlying the antibiofilm potential of curcumin against *P. aeruginosa* is through iron acquisition and interference in the metabolic intermediates of virulence factor production. Similar antibiofilm activities have been reported for *Acinetobacter baumannii* [121], *L. monocytogenes* [122], *Enterococcus fecalis* [123] and other organisms. Additionally, curcumin exerted antibiofilm activity against mixed species biofilms of *C. albicans* and *S. aureus* [124]. These reports suggest that curcumin is a potential antibiofilm agent in treating biofilm-associated infections.

### 3.5. Bioactive Efficacy of Curcumin against Human Diseases and Metabolic Disorders

In recent decades, curcumin has been well recognized for its versatile therapeutic properties. Curcumin has also been shown to have promising pharmacological activities against common human diseases such as arthritis, neuroprotective activity, hepatoprotective effect, cardioprotective activity and metabolic syndrome [125]. The influence of curcumin on various human diseases is briefly discussed below.

#### 3.5.1. Influence of Curcumin on Neurodegenerative Disorders

Alzheimer’s disease, Huntington’s disease, Parkinson’s disease, spinal muscular atrophy, motor neuron disease and Friedreich’s ataxia are very common neurodegenerative disorders found worldwide. Neurodegenerative disorders are serious and life-threatening to the human population, and they affect most of the regular activities of humans, such as movement, talking, breathing and other biological functions [126,127]. Most of the neurodegenerative disorders are caused by genetic factors and other human activities, including alcoholism, prolonged medication, toxic substances, smoking, etc. Most of the available treatments for neurodegenerative disorders may relieve pain and reduce the impact of disease. There is no complete recovery treatment against most of the neurodegenerative disorders [128]. Alzheimer’s disease is a progressive neurodegenerative disorder that continuously abolishes human memory power and regular physical activity. Amyloid-β plaques formation is the main histopathological reason for Alzheimer’s disease. Alzheimer’s disease occurs due to formation of amyloidal plaques in the nerve cells owing to cleavage of amyloid precursor proteins into amyloid-beta (Aβ) peptides through proteolytic processing using secretase enzymes including β and γ-secretase. Also, neuroinflammation, neurofibrillary tangle formation, accumulation of hyperphosphorylated tau proteins, vascular abnormalities, oxidative stress and mitochondrial damage are the reasons for Alzheimer’s disease [129]. Currently, pharmacological-based therapy to prevent or cure Alzheimer’s disease is very meagre. However, clinically approved drugs are available for the treatment of Alzheimer’s disease, such as donepezil, maypostpone and galantamine. These drugs have shown temporary efficacy against Alzheimer’s disease and have also been reported for their side effects [130]. Several reports have revealed that bioactive compounds from natural sources could show potential activity against Alzheimer’s disease. For instance, Utomo et al. [131] developed a curcumin-based SAR matrix that has a great inhibitory effect on amyloid-β aggregation to treat Alzheimer’s disease. This study also unveiled the non-cytotoxicity effect of a curcumin-based SAR matrix and reduced Aβ-induced cytotoxicity. Finally, their study conclude that a curcumin-based SAR matrix has the ability to prevent the neurotoxicity which causes Alzheimer’s disease [131]. Kim et al. [132] reported that curcumin impedes oxidative stress generated due to Aβ and it helps to decrease the number of oxidized proteins and inflammatory cytokines.

Parkinson’s disease is one of the notable neurodegenerative disorders and it is clinically categorized as a movement disorder associated with age and genetic factors of the human population. This disease arises due to the degeneration of particular dopaminergic neurons in the substantia nigra of the ventral midbrain, through which it reduces the level of dopamine in the striatum [133]. All the emerging treatments for Parkinson’s disease are intended to increase the dopamine level. Further, drugs used for the treatment of Parkinson’s disease do not show strong neuroprotective effects. Subsequently, novel studies have revealed that natural molecules have neuroprotective activity in the treatment of Parkinson’s disease [134]. Curcumin is one of the natural compounds identified with neuroprotective activity. Numerous studies have been conducted to investigate the clinical application, therapeutic potential and potent activity of curcumin against Parkinson’s disease [135]. Nguyen et al. [136] reported that curcumin treatment improves locomotive abilities and induces less severe neurodegeneration in the Drosophila melanogaster model (dUCH knockdown) for Parkinson’s disease. Furthermore, Sharma and Nehru [137] evaluated the inhibitory potential of curcumin against alpha-synuclein (one of the crucial proteins responsible for Parkinson’s disease) by generating oxidative stresses, including hydrogen peroxide, dopamine oxidation, nitric oxide synthase, glutathione depletion and NADPH oxidase activation. Surprisingly, epidemiological reports stated that the frequency of Alzheimer’s disease and Parkinson’s disease is very low in the Indian population when compared to the Europeans and this may be due to the enormous consumption of turmeric and curcumin by Indians [138].

#### 3.5.2. Hepatoprotective Activity of Curcumin

Globally, hepatic diseases are one of main causes of mortality and morbidity among the human population. There are several factors that induce hepatic dysfunctions, such as drugs, toxic substances, microbial infections, medications, oxidative stress, inflammation, chronic alcoholism and human modern habits [139]. There are numerous plant-associated compounds explored with potent hepatoprotective activities, among which curcumin is one of the well-known plant-derived bioactive compounds that can be used as a significant compound source for the development of hepatoprotective drugs [140]. A study by Girish and Pradhan determines the ability of curcumin to improve liver cell regeneration in mice by restoring the cytochrome P450 enzyme system and reducing oxidative stress [141]. Further, El Swefy et al. [142] demonstrated that curcumin enhanced biochemical and structural properties of liver cells affected by bile duct ligation-induced fibrosis. Generally, the hepatoprotective efficacy of curcumin is observed through inhibiting COX-2, NF-κB, lipid peroxidation and reduced glutathione. Thus, the mechanisms driving the hepatoprotective activity of curcumin were through the inhibition of oxidative stress and inflammation in the liver. Curcumin, as a result, plays a significant therapeutic role in the treatment of liver diseases [143].

#### 3.5.3. Efficacy of Curcumin against Arthritis

Arthritis is one of the autoimmune disorders caused by hyperplasia of the cartilage lining. It is observed due to the accumulation of inflammatory cells in synovial tissue, which leads to joint damage, significant pain and disability. Several cytokines are responsible for arthritis, such as IL-1b, TNF-α, IL-1β, IL-6 and IFN-γ of dendritic cells, T cells and macrophages, respectively [144]. Thus, inhibition of these pro-inflammatory cytokines may be a strong therapeutic strategy for the treatment of arthritis. The current pharmaceutical drugs for treating arthritis are analgesics, ion nonsteroidal anti-inflammatory drugs, steroids, disease-modifying antirheumatic drugs and nonsteroidal anti-inflammatory drugs. They have the potential to reduce the symptoms of severe pain and inflammation. However, these drugs are responsible for causing numerous serious side-effects, including insufficient pain relief, gastrointestinal disorders, immune system disturbances and cardiovascular adverse effects [145,146]. Among the explored bioactives, curcumin is one of the notable bioactive compounds that could be used against arthritis due to its anti-inflammatory property. Moon et al. [147] reported that curcumin treatment improves recovery of collagen-induced arthritis in mice and IL-1β-induced activation in fibroblast-like synoviocytes. In addition, arthritis-associated factors, such as TNF-α, IL-1β, NF-κB transcription activity, prostaglandin E2, matrix metalloproteinases and COX-2 expression were inhibited by curcumin treatment. Several studies investigated the therapeutic potential of curcumin against arthritis by targeting the pro-inflammatory cytokine responsible for arthritis [148,149,150].

#### 3.5.4. Attenuation of Metabolic Syndrome by Curcumin

Metabolic syndrome is a disease caused by multiple factors such as aging, genetics and human lifestyle, including physical inactivity, obesity, inappropriate nutrition, etc. It is assessed through the following criteria: high-density lipoprotein cholesterol, fasting plasma glucose, triglycerides, blood pressure and obesity [151]. Curcumin has attenuated the various traits of metabolic syndrome by reducing high-density lipoprotein cholesterol, inflammation, oxidative stress, blood pressure and improving insulin sensitivity [152]. Furthermore, there are reports available for curcumin altering the lipoprotein metabolism, which is responsible for plasma triglycerides, cholesterol and high-density lipoprotein cholesterol quantity, thereby reducing the obesity-associated metabolic syndrome [153]. Pro-inflammatory cytokines have been linked to metabolic syndrome, which includes obesity, diabetes and cardiovascular disease. There, curcumin helps recovery from various metabolic syndrome-associated pro-inflammatory cytokines [5]. A study reported that curcumin supplementation to patients with metabolic syndrome-related disorders reduced weight and leptin levels while increasing adiponectin levels [154].

### 3.6. Wound Dressing Application

A wound is an injury that damages the physical structure of the skin and results in fluid loss and pain. The healing of wounds is a natural phenomenon that involves various cellular and molecular processes. Numerous biological properties, such as anti-inflammatory, antioxidant and antibacterial activity, make curcumin a potential wound healing agent. Oxidative stress is the major cause of delayed wound healing and makes the wound chronic. A previous study proved that reduction of ROS at the site of injury accelerates the healing process. Hence, targeting ROS was speculated to be the mechanism of curcumin to enhance wound healing [155]. As an antioxidant agent, curcumin has effectively enhanced the wound healing process by eradicating ROS, as demonstrated by in vitro and in vivo experiments. In addition, curcumin also decreases the expression of inflammatory factors to reduce the inflammatory damage of local tissue and thereby supports wound healing [156].

Curcumin has been widely explored for wound healing applications not only because of its therapeutic activities, but also for its non-toxic nature. Curcumin is designated as a “generally regarded as safe” (GRAS) compound by the U.S. Food and Drug Administration (FDA). Hence, it is not surprising that curcumin is globally employed for biomedical applications [15]. However, the hydrophobic nature of curcumin results in poor solubility in the aqueous phase and limits the bioavailability [157]. Many polymer-based wound dressings are being tested to overcome these limiting factors. Topical application of curcumin is predicted to be the best way of administration to avoid the bioavailability issues associated with systemic mode. Table 3 summarizes the various formulations loaded with curcumin for wound dressing application.

## 4. Bioavailability of Curcumin: Problems and Prospects

Regardless of its potential advantages, curcumin has not been efficient in treating a variety of ailments due to its low bioavailability, which is characteristic of a weak and impractical drug. Low solubility, limited gastrointestinal tract absorption, quick metabolism, rapid systemic clearance and restricted blood–brain barrier (BBB) penetration are all major factors that contribute to the low bioavailability [176]. Orally consumed curcumin is rapidly conjugated in the small intestine, liver and kidneys into curcumin glucuronide, curcumin sulphate and methylated curcumins, which are rapidly excreted in the urine and feces [177,178]. Similarly, if administered systemically, it turns into tetrahydrocurcumin, hexahydrocurcumin and octahydrocurcumin [179]. Curcumin is largely found in the blood as physiologically and pharmacologically inert conjugates, with comparatively little free bioactive curcumin, comparable to other polyphenol compounds. Furthermore, when compared to other known drugs, various researchers have revealed that curcumin has unfavorable pharmacokinetics in terms of absorption, distribution, metabolism, excretion and toxicity (ADMET) properties [180]. It is also unstable in aqueous solutions due to its hydrophobic nature, and it degrades promptly at physiological pH [181].

However, the most challenging factor is low water solubility (0.4 μg/mL at normal pH). Furthermore, in a clinical trial study of curcumin on Alzheimer’s disease (AD), no difference in absorption was found between the 1 g and 4 g doses. As per a previous report, curcumin and its metabolites were not discovered in the blood after administration due to its restricted tissue dispersion [182]. In a report of a clinical trial, patients with an advanced stage of colorectal cancer were given 440–2200 mg/day of oral curcumin extract for 29 days. Curcumin and its metabolites were not detected in the plasma or urine of patients [183]. Similarly, following an oral dose of 8.0 g of curcumin per day, only 22–41 ng/mL of curcumin was measured in plasma in a Phase II trial of curcumin therapy for patients with advanced pancreatic cancer [184].

**Figure 6:**
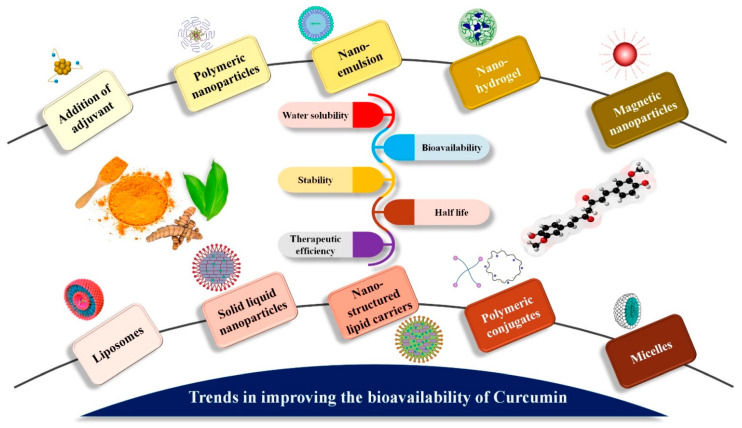
Trends in enhancing the oral bioavailability of curcumin. As shown in the image, various methods of nanotechnology have been used to enhance bioavailability of curcumin.

Regardless, curcumin’s substantial limitations have not stopped scientists from researching and enhancing its potential. Rather, they have created greater scope for the development of innovative solutions to address issues with the native form. Curcumin formulations have come about because of research to improve bioavailability, permeability, circulation and half-life, as well as withstand metabolic processes. These formulations admit chemical curcumin derivatives and analogs with metabolic adjuvants, nanoparticles, liposomes, micelles, nanostructured lipid carriers (NLC) and phospholipid complexes [180] (Figure 6). Phospholipid complexes appear to have been effectively released into the worldwide market among the formulations that emerged (Meriva^TM^ and Longvida^TM^). Longvida^®^ (Verdure Sciences, Noblesville, IN, USA), the bioavailability of a solid lipid formulation containing roughly 80 mg of curcumin, was observed to be four times better than that of curcumin alone. In an aged population, daily therapy with 400 mg of Longvida^®^ for four weeks resulted in better cognitive capabilities [185]. Meriva^®^ (Indena, Milan, Italy), another proprietary curcumin formulation from Indena, is used to treat osteoarthritis, diabetes mellitus and microangiopathy in a 1:2 weight ratio of curcumin and soy lecithin. In comparison to Meriva, normal curcuminoid combination absorption was 29-fold lower in clinical studies [186]. When compared to C95, which contained 95% of curcuminoid powder (curcumin, demethoxycurcumin and bisdemethoxycurcumin) and micronized curcuminoids, plus turmeric oil, showed five times improvement in bioavailability [187]. The CCC (curcumin cyclodextrin complex) is said to have a 45-fold higher bioavailability than C95 [188]. CPC (curcuminoid phospholipid complex) demonstrated a 20-fold better bioavailability when compared to curcumin alone and 30-fold higher than total curcuminoids [187]. Another study by Zeng et al. [189] examined the effect of piperine pre-administration on oral curcumin bioavailability. In this investigation, rats were given 20 mg/kg piperine first, followed by 200 mg/kg curcumin at intervals of 0.5 to 8 h after piperine treatment. Piperine pre-treatment before curcumin administration resulted in a significant increase in curcumin oral bioavailability in all tested rats. Curcumin permeability rose 1.85-fold when quercetin and resveratrol were combined. This suggested that resveratrol and quercetin had a cumulative impact on curcumin absorption [190].

## 5. Recent Trends to Improve Oral Bioavailability of Curcumin

### 5.1. Use of Adjuvants

Though curcumin has a lot of advantages, a large number of preclinical studies evidenced that it cannot be readily used to treat any diseases in human beings owing to its incredibly poor bioavailability [191]. One of the important strategies to improve the oral bioavailability of curcumin is using the adjuvants to neutralize detoxification enzymes demonstrated in the curcumin metabolism. Among the adjuvants, piperine (a compound from black pepper) is found to be one of the greatest enhancers of curcumin bioavailability [192]. Serum concentration was reported to be increased by the concomitant administration of curcumin with piperine in humans/animals (more than a thousandfold) [193]. Additionally, epigallocatechin-3-gallate (EGCG) was used as an adjuvant to curcumin which could improve the curcumin bioavailability to many folds [194]. The adjuvants are not limited to these above-mentioned compounds only, however. A number of adjuvants have been used to increase the oral bioavailability of curcumin for various therapeutic applications, including cancer. A recent article reported that oral administration of curcumin along with piperine for COVID-19 treatment (symptomatic) could significantly decrease mortality and morbidity [195]. Also, the article represents that curcumin, along with piperine, may be a natural therapeutic and safe choice to prevent post-COVID thromboembolic measures as well. One more research article emphasized that sustained topical delivery and enhanced bioavailability of curcumin was achieved with curcumin nanoparticle, notably the potential for skin staining. A murine model organism was used to investigate the skin inflammation with or without ultraviolet-B radiation exposure along with encapsulated/unencapsulated curcumin in coconut oil. After 24 h incubation, the experimental setup treated with encapsulated curcumin showed less skin reddening than those treated with only curcumin. Also, inflammation cytokine analysis and histology of the encapsulated curcumin-exposed skin exhibited less skin cell damage and decreased inflammation (markers) when compared to control groups and nonencapsulated groups [196]. Recently, evidence suggested that nanocapsulated/unencapsulated forms of curcumin functions as an effective bio molecule for restoring the host–microbial relationships and healthy gut homeostasis [197,198]. However, it is suggested that detailed research investigation at the preclinical/clinical stage is mandatory to explore the full potential of the adjuvant role of curcumin and other bioactive components in various applications, including cancer prevention and treatment [199].

### 5.2. Nanoformulation of Curcumin: Nanoparticle, Nanocomposite, Hydrogels, etc.

The nanoformulation of curcumin has been demonstrated with vast clinical applicability to utilize the real potential of the ultimate drug candidate, curcumin. The major nanoformulations of curcumin include nanoemulsions, solid lipid nanoparticles, nanocomposite, nanosuspension, nanoparticles, liposomes, micelles, polymeric nanoparticles, hydrogels, etc. [200]. Liposomes are vesicular structures with one or more phospholipid bilayers capable of delivering potential drug molecules to the cells of interest. Liposomes bind to the target cell’s lipid membrane and release the liposomal substance into the cytoplasm. Liposomes may entrap both lipophilic and hydrophilic molecules, ensuring optimal effectiveness and safety in the delivery of the target-specific medication [201]. Micelles are core–shell nanostructures with a lipophilic core and a hydrophilic outer layer that make up the shell. They are made by amphiphilic co-polymers self-assembling at a crucial micellar concentration. The hydrophobic core serves as a good carrier for drugs that are hydrophobic [202]. Nanosuspensions are water-insoluble pharmaceuticals dispersed in aqueous solutions without the need for a carrier. The medications have a colloidal size range of less than 1 µm and surfactants and other substances incorporated as stabilizers. The combination of tiny particle size (PS) and a large surface area, as well as high thermodynamic energy, favors fast drug dispersion [167]. Solid lipid nanoparticles (SLNs) are biocompatible and biodegradable colloidal lipid carriers (50–1000 nm) composed of biological lipids. Unlike liposomes, SLNs are hard particles that are only suited for loading hydrophobic compounds such as curcumin. High drug-loading capacity, strong stability, outstanding biocompatibility and increased bioavailability are all prominent attributes of SLNs. Being hydrophobic, they are effective nanocarriers for controlled release and site/cell-directed medication administration to the reticuloendothelial system [203].

Nanoemulsions (NEs) are clear or translucent dispersions of oil, emulsifier and water with particle sizes within 100 nm that are kinetically stable. In contrast to microemulsions, NEs do not develop spontaneously; generating NEs requires a significant amount of energy due to the low surfactant content in them. As they are emulsions, they allow for the inclusion of both hydrophobic and hydrophilic therapeutics, as well as the bio-enhancement of hydrophobic pharmaceuticals due to very small vesicle size [204].

Nano-sized quantum dots, manganese phosphate nanoparticles, noble metals, carbon nanotubes, silica nanoparticles and magnetic nanoparticles are examples of inorganic nanoparticles. They have unique physical characteristics that are size-dependent, including optical and electrical effects, efficient contrasting effect and magnetism. Additionally, they are resistant to microbes and have strong storage characteristics. Curcumin inorganic NPs have the potential to be employed in a variety of essential bio-applications [205]. Polymeric nanoparticles are granular colloids with a diameter of up to 1000 nm that are made from natural or synthetic polymers that are biodegradable or not. They are classified as either nanocapsules (where the medication is contained within a cavity covered by a polymer covering) or nanospheres (where the medication is distributed throughout the polymer). When compared to liposomes, polymeric NPs have more reactivity, surface area, sensitivity and stability. They are engaging drug carriers because of their high membrane permeability (due to their small size) and their ability to target specific organs [206]. A list of previously published curcumin nanoformulations with various medical applications is shown in Table 4.

Nanocomposites are non-homogenous materials made at the nanoscale level by combining polymers with inorganic solids. Their frameworks have been discovered to be more complex than microcomposites. Individual property structure, composition, interfacial interactions and components have a significant impact on them. The method of in situ growth and polymerization of biopolymer and inorganic matrix is the most common way to create nanocomposites [222].

Hydrogels are three-dimensional (3D) polymeric structures that are extremely swollen, hydrophilic and capable of absorbing huge volumes of water insoluble in water, due to cross-linked polymers, enmeshment or crystalline areas in its composition. Natural, synthetic or hybrid polymers can be used to make hydrogels. Biopolymer-based hydrogels have attracted a lot of attention as promising options for medicinal applications, including therapeutics delivery [223]. As a result, nanotechnology has been shown to be a very effective tool to increase the limits of native curcumin for the improvement of its therapeutic potential in combination with some key features such as high cellular uptake, bio-distribution, dissociation rates, stability in serum and sustained drug release at the target site. 

### 5.3. Structural Analogs

Typically, curcumin analogs are divided into three groups, namely, natural derivatives from turmeric, curcumin analogs from mother nature and synthetic analogs [224] (Table 5). Curcumin, cyclocurcumin, bisdemethoxycurcumin and demethoxycurcumin (generally referred as curcuminoids) are the three major analogs originated from turmeric. Bioactive compounds that occur naturally and also possess structural similarity to curcumin are defined as curcumin analogs from mother nature. Synthetic analogs of curcumin compounds are produced by modifying the basic structure of curcumin using various chemical reactions. The major possibilities to modify curcumin structurally are making changes in the dike to functionality, aryl side chain, active methylene functionality, double bond and metal complexes [225]. Some of the synthetic analogs are EF24 [226], (1E, 6Z) 1,7-bis(13-fluoro-9-ethylcoumarin-8-yl)-5-hydroxy3-oxohepta-1,4,6-triene [227].

### 5.4. Liposomal Curcumin

Curcumin is likely to be metabolized quickly with less photo-stability, making it an ineffective therapeutic agent in its natural form [236]. In this context, various reports suggest that these restrictions can be reverted by solubilizing the curcumin in a phospholipid bilayer, liposomes, etc., which can facilitate the delivery/bioavailability of curcumin with ease [237]. It is well known that nanoparticles (liposomes, micelles, etc.) have been considered to improve the intracellular and targeted drug delivery of curcumin, which can cross the inaccessible anatomical and physiological barriers. Thus, liposomes must be a successful method to increase the bioavailability and stability of curcumin [238]. Major preparation methods to produce curcumin liposomes include an injection method, a reversed-phase evaporation method, thin-film method, freeze–thawing method, etc., [239]. Basically, curcumin encapsulation including liposomes for any type of cancer/disease should be of enhanced bioavailability when compared to freely available curcumin. At the same time, it must be non-toxic in nature to normal cells in the resulting environment. In this context, the synthesis of liposomes can be done using a solvent-free method as well (since solvents may be toxic). For instance, De Leo et al. [240] reported the encapsulation method of curcumin liposomes for drug delivery in pH-responsive polymers using an organic free method. It is reported that liposomal curcumin was found to have enhanced pharmacological and antitumor activities put forth by improving the pharmacodynamics and pharmacokinetics. Especially, liposomal curcumin was prepared with diverse conjugates including vitamin A, polyethylene glycol, hyaluronic acid, silica and folic acid. Besides, liposomal nanoparticles encapsulated with drug combinations could sensitize cancer cells in the OS cell line (human osteosarcoma cell line) [241]. However, with the constant progress of various liposomes, curcumin liposomes should be more standardized for other diseases as well as cancer.

### 5.5. Curcumin Phospholipid Complex

Several studies have implied the crucial roles of phospholipids in improving the therapeutic efficiency of small molecules for those who have poor oral bioavailability [242]. Generally, amphipathic phospholipid complexes act as bioactive components to aid in passing them through the gastrointestinal cells to the blood eventually [85]. Theoretically, phospholipid complexes are explained as appropriate strategies for any bioactive small molecule. The curcumin molecule is found to have high affinity towards biological membranes and tends to penetrate them rapidly to form dimeric biological complexes. Despite being a phenolic and poorly soluble compound, curcumin can link with phospholipids (particularly phosphatidylcholine) by forming non-covalent adducts. At last, formation of these curcumin–phospholipid complexes could enhance the curcumin pharmacokinetics by stabilizing at intestinal pH values and shielding curcumin in terms of retro-Claisen hydrolysis [243,244]. Maiti et al. [245] reported that a curcumin–phospholipid complex enhanced the oral bioavailability of curcumin when compared with curcumin suspension to five folds. In another study, results of ex vivo absorption of a phospholipid–curcumin complex in Wistar rats showed that the bioavailability was significantly more enhanced than the kinetically free curcumin. In addition, pharmacokinetic study results revealed that a phospholipid–curcumin complex implied significantly high plasma concentrations and was found to be more stable when compared to natural curcumin [246]. There are various research findings that suggested the phospholipid–curcumin complex is one of the most precious methods for making the curcumin more stable and improving its bioavailability [191,247].

## 6. Conclusions

Curcumin, the natural polyphenol of the turmeric rhizome, is a food additive, food colorant and natural dye that is also known for its huge spectrum of therapeutic applications and has been practically used in traditional medication for decades. In addition, a relatively low dose of curcumin can provide various health benefits for people diagnosed with any health condition. In the recent past, several studies have been involved in increasing the therapeutic efficiency and clinical applications of curcumin in order to improve the treatment of various diseases and disorders. Yet, due to poor solubility and low bioavailability, the direct usage of curcumin in clinical applications is challenging. With the advent of new technologies and the introduction of various alternative therapeutic approaches, these drawbacks have also been addressed. As of today, synthetic derivatives and various nanoforms of curcumin are being utilized in several biomedical applications, large scale production, stability, bioavailability, quality control of drug loading, etc. This review illustrated the role of curcumin in terms of structure and biochemical properties, as well as mechanisms of action, in order to aid in understanding its significant therapeutic applications and valuable health benefits. Further, we elaborated on the hurdles, recent trends and future perspectives on improving the bioavailability of curcumin for the effective utilization of curcumin in biomedical applications.

## 7. Future Perspectives

Additional validation studies are enormously needed to address and investigate the clinical effectiveness of both free curcumin and its analogs in various human diseases, including cancer, neurodegenerative disease, etc. The important gaps identified in using curcumin for therapeutic applications and its effectiveness include a complete safety/toxicity profile and data-dependent analysis. Additionally, there is less information available relating to the nanocarriers and a suitable delivery system for curcumin for increased targeted and therapeutic applications. More research needs to be undertaken to identify the appropriate formulation and delivery system for each of the biomedical application. Furthermore, clinical trials need to be conducted to analyze the increased therapeutic efficiency of the nanoform of curcumin, structural analogs and their delivery techniques. Following research to fill these knowledge gaps can improve the magnitude and competence of curcumin in various clinical applications. In addition to this, identifying the molecular targets of curcumin and their mechanisms of action against various human diseases will help in developing more specific structural molecules that can exert precise remedial activity against the disease under study. That said, improved oral bioavailability and convincing preclinical/clinical trials of curcumin are more likely to bring this promising natural small molecule to the cutting edge by creating a “super curcumin molecule” quite soon.

## Figures and Tables

**Table 1:** Physicochemical properties of curcumin.

Formula	C_21_H_20_O_6_
Molecular weight	368.38 g/mol
Num. heavy atoms	27
Num. arom. heavy atoms	12
Fraction Csp3	0.14
Num. rotatable bonds	8
Num. H-bond acceptors	6
Num. H-bond donors	2
Molar refractivity	102.80
Topological polar surface area	93.06 Å^2^
Nviolation	0
Melting temperature	176–177 °C
cLogP	3.2
cLogS	−3.94
Bioavailability score	0.55
Gastrointestinal absorption	High
Blood–brain barrier (BBB) permeant	No

**Table 2:** Biological activities and their molecular mechanisms of curcumin.

S. No.	Biological Activities	Molecular Mechanisms	References
1	Antioxidant	Scavenges the free radicals (ROS, RNS); Obstructs the enzyme (GSH, catalase, GPx and SOD) activity to optimize the free radicals; Alters the ROS-producing enzymes such as lipoxygenase (LOX)/cyclooxygenase (COX), xanthine oxidase.	[25,26,27,28]
2	Anticancer	STAT3 and NF-κB signaling pathways play a crucial role in cancer growth. Curcumin eradicates or obstructs its growth. It controls the migration and invasion by directing the expression of Sp-1 and housekeeping genes.	[29,30,31]
3	Antimicrobial	Interacts with FtsZ, which is a significant player in cell division initiating proteins.	[32,33,34]
4	Anti-inflammatory	Regulates and controls the inflammatory response via decreasing the enzymatic activity of phospholipases A2 (PLA2s), lipoxygenase (LOX) and cyclooxygenase-2(COX-2), triggering the nitric oxide synthase (iNOS) enzymatic pathways that can modulate prostaglandin synthesis and essential inflammatory response mediators.	[35,36,37]
5	Antifibrosis	Prevents the migration and proliferation abilities of fibroblast and collagen production via altering the expression of transforming growth factor (TGF)-β and angiotensin signaling (Ang).	[38]
6	Antiamyloid	Regulates amyloid beta (Aβ) metabolism; Inhibits Aβ aggregation; Disaggregates to form fibrillar Aβ16, Aβ40 and Aβ42 to produce strong anti-amyloidogenic effects.	[39,40]
7	Antidiabetic	Suppresses the advanced glycation end products (AGEs) formation; Stimulates peroxisome proliferator-activated receptor gamma (PPARg) activity; Increases glutathione synthesis; Increases secretion of insulin from pancreatic cells, as well as reduces insulin resistance.	[41,42]

**Table 3:** Various formulations loaded with curcumin for wound healing application.

Type of Formulation	Composition	Reference
Films	Methoxy PEG-g-chitosan composite films encapsulated with curcumin	[158]
	PLA/chitosan nanofilms loaded with curcumin	[159]
	Antimicrobial cellulose nanocrystals film loaded with curcumin	[160]
	Carboxymethylated guar gum-g-gelatin film encapsulated with curcumin	[161]
	Cellulose/chitosan microcrystals antimicrobial films loaded with curcumin	[162]
Hydrogel	Cellulose nanofiber-PVA hydrogels incorporated with curcumin	[163]
	Thermal-responsive hydrogels from chitosan–Pluronic P123 loaded with curcumin and gelatin	[164]
	Cellulose–halloysite nanotube hydrogel loaded with curcumin	[165]
	Nanosilver nanohydrogels of polymethacrylic acid blended with a PEO/PVA/carboxymethyl cellulose matrix and loaded with curcumin and aloe vera	[166]
	PEG-poly (ε-caprolactone) (PCL)-PEG copolymer-based in situ gel-forming hydrogels loaded with curcumin	[167]
	Thermosensitive β-glycerophosphate/chitosan hydrogels encapsulated with β-cyclodextrin-curcumin	[168]
Bandages	Oleic acid-based chitosan–alginate bandages loaded with curcumin	[169]
	Chitosan–alginate bandages co-encapsulated with curcumin	[170]
Nanofibers	PCL-based nanofibers loaded with curcumin	[171]
	Curcumin-loaded PLA nanofibers	[172]
	Curcumin-loaded PLA/PEG nanofibers	[173]
	PCL-PEG nanofibers incorporated with curcumin	[174]
	Electrospun poly (3-hydroxybutyric acid-co-3-hydroxyvaleric acid) (PHBV)-based nanofibers loaded with curcumin	[175]

**Table 4:** Name list of the nanoformulations and their corresponding therapeutic applications.

S. No.	Name of the Nanoformulation	Formulation Ingredients and Method	Application	References
1	Cur@CRN	Nanocomposites containing Cur and ι-Carrageenan using emulsion technique	Enhanced stability, oral bioavailability, antioxidant and anti-inflammatory effects	[207]
2	CUR-Gel-HAp	Encapsulation of curcumin into the hydrophilic network of gelatin (Gel) and hydroxyapatite (HAp) by colloidal suspension	Enhanced cytotoxicity towards human lung cancer cells (A549)	[9]
3	Nanocurcumin	Nanomicelle with an average size of 50 nm containing curcumin	Enhanced antioxidant and anti-inflammatory effects against LPS-induced coliform mastitis in rat model	[208]
4	Curcumin nanoparticle	Curcumin loaded in poly lactic-*co*-glycolic acid nanoparticles with an average size of ~90 ± 10 nm by green surfactant-based synthesis	Enhanced solubility, photo-stability, antibiofilm activity and cytotoxicity towards HepG2 cells	[209]
5	CUR-loaded NC	Curcumin-loaded polymeric nanocapsules	Enhanced antiangiogenic, non-teratogenic and antioxidant effects in chick embryo model.	[210]
6	MSN-HA-C	Curcumin loaded in nanohybrid composed of mesoporous silica nanoparticle (MSN) and hyaluronic acid (HA) with an average size of ~75 nm	In vivo and in vitro anti-breast cancer activity	[211]
7	Fe@Au-CU-CS-FA NP’s	Nanoencapsulation comprised of folic acid, gold, curcumin, chitosan and iron synthesised by microemulsion method	Cytotoxic effect on lung cancer cells	[212]
8	SD-CUR	A core–shell solid dispersion composed of curcumin-loaded micelles self-assembled from disodium glycyrrhizin (Na2GA), coated with pectin and tannic acid	Enhanced bioavailability and antihyperlipidemic activity	[213]
9	SN_LYZ_-Cur	The nanoconjugate SN_LYZ_-Cur composed of curcumin surface-conjugated on self-assembled lysozyme nanoparticle with an average size of ~120 nm	Antibacterial, antioxidant and anticancer activity against multiple cancer cell lines such as HeLa, MCF-7, MDAMB-231 and MG 63	[214]
10	Cur-CasNPs	Encapsulation of curcumin in casein nanoparticles	Enhanced bioavailability and anticancer activity against MCF-7 cell lines	[215]
11	CDD-CANPs	Encapsulation of curcumin diethyl disuccinate (CDD) in chitosan–alginate nanoparticles (CANPs)	High chemical stability upon UV light exposure, fivefold enhanced bioavailability and anticancer activity against HepG2 cell lines	[216]
12	Cur NE-CLA-n-3FA	Nanoemulsions containing curcumin stabilized with mono- and diacylglycerols structured with conjugated linoleic acid (CLA) and n-3 fatty acids (n-3FA)	Greater bioavailability	[217]
13	Cur-C3-CNPs	Nanoparticle composed of curcumin-C3 complex (curcumin, demethoxycurcumin and isdemethoxycurcumin) encapsulated in chitosan	Antioxidant and antibacterial activity	[12]
14	CLL	Nanoliposomes (formulated from salmon, rapeseed and soya lecithin) comprising of curcumin encapsulated in chitosan	Increased bioavailability and anticancer activity against MCF-7 breast cancer cell lines	[218]
15	Cur-BR liposome	Nanoliposome containing curcumin and bromocriptine synthesized by thin-film method	In vitro anticancer activity against lung cancer cells by induction of apoptosis	[219]
16	LRA-CS-CUR hydrogel	A composite hydrogel containing lotus root amylopectin (LRA)-chitosan (CS) in the ratio of 3:2 at pH 4.0 with an average size of 410.3 nm	Enhanced stability in stomach and sustained release in small intestine	[220]
17	Chitosan-PEG-Cur-hydrogel	Hydrogel comprising of chitosan, polyethylene glycol and curcumin using microwave technology at frequency of 2450 MHz, 500 Watt and 120 s time.	In vivo open incision wound healing activity	[221]

**Table 5:** List of natural derivatives and analogs of curcumin.

Compound Name	Origin		PubChem CID	Molecular Formula	References
	Scientific Name	Common Name			
Curcumin natural derivatives from turmeric					
Curcumin	*Curcuma longa*	Turmeric	969516	C_21_H_20_O_6_	[228]
Bisdemethoxycurcumin	*Curcuma longa*	Turmeric	5315472	C_19_H_16_O_4_	[229]
Cyclocurcumin	*Curcuma longa*	Turmeric	69879809	C_21_H_20_O_6_	[230]
Demethoxycurcumin	*Curcuma longa*	Turmeric	5469424	C_20_H_18_O_5_	[231]
Curcumin natural analogs from mother nature					
Dehydrozingerone	*Zingiber officinale Roscoe*	Ginger	5354238	C_11_H_12_O_3_	[232]
Cassumunin B	*Zingiber cassumunar*	Ginger	10054109	C_34_H_36_O_9_	[224]
Cassumunin A	*Zingiber cassumunar*	Ginger	10460395	C_33_H_34_O_8_	[224]
6-shogaol	*Zingiber officinale*	Ginger	5281794	C_17_H_24_O_3_	[233]
6-paradol	*Zingiber officinale Roscoe*	Ginger	94378	C_17_H_26_O_3_	[234]
6-gingerol	*Zingiber officinale Roscoe*	Ginger	442793	C_17_H_26_O_4_	[235]

## Supplementary Materials

The following are available online at https://www.mdpi.com/article/10.3390/pharmaceutics13122102/s1, Table S1: Molecular targets of Curcumin and its details.
