# Supplementary material for: Biomedical Applications and Bioavailability of Curcumin—An Updated Overview
Source: Pharmaceutics. 2021 Dec 7;13(12):2102. doi: 10.3390/pharmaceutics13122102 (PMC8703330; doi:10.3390/pharmaceutics13122102)
Supplement: Supplementary file 1 [file pharmaceutics-13-02102-s001.zip › pharmaceutics-1477231-supplementary.pdf]

pharmaceutics

MDPI

Supplementary Materials: Biomedical Applications and Bioavailability of Curcumin—An Updated Overview

Soo-In Sohn, Arumugam Priya, Boopathi Balasubramaniam, Pandiyan Muthuramalingam, Chandran Sivasankar, Anthonymuthu Selvaraj, Alaguvel Valliammai, Ravi Jothi and Subramani Pandian

Table S1. Molecular targets of Curcumin and its details.

Target	Common name	Uniprot ID	ChEMBL ID	Target Class
Monoamine oxidase A	MAOA	P21397	CHEMBL1951	Oxidoreductase
Beta amyloid A4 protein	APP	P05067	CHEMBL2487	Membrane receptor
Histone acetyltransferase p300	EP300	Q09472	CHEMBL3784	Writer
Prostaglandin E synthase	PTGES	O14684	CHEMBL5658	Enzyme
Toll-like receptor (TLR7/TLR9)	TLR9	Q9NR96	CHEMBL5804	Toll-like and Il-1 receptors
Beta-secretase 1	BACE1	P56817	CHEMBL4822	Protease
DNA topoisomerase II alpha	TOP2A	P11388	CHEMBL1806	Isomerase
Glyoxalase I	GLO1	Q04760	CHEMBL2424	Enzyme
Nuclear factor erythroid 2-related factor 2	NFE2L2	Q16236	CHEMBL1075094	Unclassified protein
Arachidonate 5-lipoxygenase	ALOX5	P09917	CHEMBL215	Oxidoreductase
Cyclooxygenase-1	PTGS1	P23219	CHEMBL221	Oxidoreductase
Inhibitor of NF-kappa-B kinase (IKK)	IKBKG IKBKB CHUK	Q9Y6K9 O14920 O15111	CHEMBL2111328	Kinase
Epidermal growth factor receptor erbB1	EGFR	P00533	CHEMBL203	Kinase
Estradiol 17-beta-dehydrogenase 3	HSD17B3	P37058	CHEMBL4234	Enzyme
Signal transducer and activator of transcription 3	STAT3	P40763	CHEMBL4026	Transcription factor
11-beta-hydroxysteroid dehydrogenase 1	HSD11B1	P28845	CHEMBL4235	Enzyme
Serine/threonine-protein kinase AKT	AKT1	P31749	CHEMBL4282	Kinase
Glycogen synthase kinase-3 beta	GSK3B	P49841	CHEMBL262	Kinase
Carbonic anhydrase VII	CA7	P43166	CHEMB	Lyase

			L2326	
Carbonic anhydrase VI	CA6	P23280	CHEMB L3025	Lyase
Carbonic anhydrase XII	CA12	O43570	CHEMB L3242	Lyase
Carbonic anhydrase XIV	CA14	Q9ULX7	CHEMB L3510	Lyase
Carbonic anhydrase IX	CA9	Q16790	CHEMB L3594	Lyase
Carbonic anhydrase VA	CA5A	P35218	CHEMB L4789	Lyase
Multidrug resistance-associated protein 1	ABCC1	P33527	CHEMB L3004	Primary active transporter
Serine/threonine-protein kinase Chk1	CHEK1	O14757	CHEMB L4630	Kinase
Pyruvate dehydrogenase kinase isoform 1	PDK1	Q15118	CHEMB L4766	Kinase
Serine/threonine-protein kinase WEE1	WEE1	P30291	CHEMB L5491	Kinase
DNA topoisomerase I	TOP1	P11387	CHEMB L1781	Isomerase
Serine/threonine-protein kinase RAF	RAF1	P04049	CHEMB L1906	Kinase
Serine/threonine-protein kinase B-raf	BRAF	P15056	CHEMB L5145	Kinase
Carbonic anhydrase II	CA2	P00918	CHEMB L205	Lyase
Carbonic anhydrase I	CA1	P00915	CHEMB L261	Lyase
Glucagon receptor	GCGR	P47871	CHEMB L1985	Family B G protein-coupled receptor
Matrix metalloproteinase 14	MMP14	P50281	CHEMB L3869	Protease
Serine/threonine-protein kinase Aurora-B	AURKB	Q96GD4	CHEMB L2185	Kinase
Plasminogen activator inhibitor-1	SERPINE1	P05121	CHEMB L3475	Secreted protein
Ribosomal protein S6 kinase 1	RPS6KB1	P23443	CHEMB L4501	Kinase
Serine/threonine-protein kinase Aurora-A	AURKA	O14965	CHEMB L4722	Kinase
Cyclin-dependent kinase 2/cyclin A	CDK2 CCNA1 CCNA2	P24941 P78396 P20248	CHEMB L2094128	Other cytosolic protein
Tyrosinase	TYR	P14679	CHEMB L1973	Oxidoreductase
Type-1 angiotensin II receptor (by homology)	AGTR1	P30556	CHEMB L227	Family A G protein-coupled receptor
NADPH oxidase 4	NOX4	Q9NPH5	CHEMB L1250375	Enzyme
Bone morphogenetic protein 1	BMP1	P13497	CHEMB L3898	Protease

Matrix metalloproteinase 13	MMP13	P45452	CHEMBL280	Protease
ADAM17	ADAM17	P78536	CHEMBL3706	Protease
Elastase 1	CELA1	Q9UNI1	CHEMBL3000	Protease
Prolyl endopeptidase	PREP	P48147	CHEMBL3202	Protease
Glutamate receptor ionotropic kainate 1	GRIK1	P39086	CHEMBL1918	Ligand-gated ion channel
Matrix metalloproteinase 8	MMP8	P22894	CHEMBL4588	Protease
Interleukin-8 receptor B	CXCR2	P25025	CHEMBL2434	Family A G protein-coupled receptor
Alkaline phosphatase, tissue-nonspecific isozyme	ALPL	P05186	CHEMBL5979	Enzyme
Maternal embryonic leucine zipper kinase	MELK	Q14680	CHEMBL4578	Kinase
Inosine-5'-monophosphate dehydrogenase 1	IMPDH1	P20839	CHEMBL1822	Oxidoreductase
Inosine-5'-monophosphate dehydrogenase 2	IMPDH2	P12268	CHEMBL2002	Oxidoreductase
Complement factor D	CFD	P00746	CHEMBL2176771	Protease
Sphingosine kinase 2	SPHK2	Q9NRA0	CHEMBL3023	Enzyme
Sphingosine kinase 1	SPHK1	Q9NYA1	CHEMBL4394	Enzyme
Mitogen-activated protein kinase kinase kinase 12	MAP3K12	Q12852	CHEMBL1908389	Enzyme
Apoptosis regulator Bcl-2	BCL2	P10415	CHEMBL4860	Other ion channel
Thyroid hormone receptor alpha	THRA	P10827	CHEMBL1860	Nuclear receptor
Thyroid hormone receptor beta-1	THRB	P10828	CHEMBL1947	Nuclear receptor
Macrophage colony stimulating factor receptor	CSF1R	P07333	CHEMBL1844	Kinase
5-lipoxygenase activating protein	ALOX5AP	P20292	CHEMBL4550	Other cytosolic protein
Histone deacetylase 3/Nuclear receptor corepressor 2 (HDAC3/NCoR2)	NCOR2 HDAC3	Q9Y618 O15379	CHEMBL2111363	Eraser
Serine/threonine-protein kinase Sgk1	SGK1	O00141	CHEMBL2343	Kinase
Dipeptidyl peptidase IV	DPP4	P27487	CHEMBL284	Protease
Dipeptidyl peptidase II	DPP7	Q9UHL4	CHEMBL3976	Protease
Heat shock protein HSP 90-beta	HSP90AB1	P08238	CHEMBL4303	Other cytosolic protein
ADAM10	ADAM10	O14672	CHEMB	Protease

			L5028	
Matrix metalloproteinase 3	MMP3	P08254	CHEMBL283	Protease
Cyclin-dependent kinase 5/CDK5 activator 1	CDK5R1 CDK5	Q15078 Q00535	CHEMBL1907600	Kinase
HERG	KCNH2	Q12809	CHEMBL240	Voltage-gated ion channel
15-hydroxyprostaglandin dehydrogenase [NAD+]	HPGD	P15428	CHEMBL1293255	Enzyme
Tyrosine-protein kinase JAK1	JAK1	P23458	CHEMBL2835	Kinase
Tyrosine-protein kinase JAK2	JAK2	O60674	CHEMBL2971	Kinase
Histone deacetylase 10	HDAC10	Q969S8	CHEMBL5103	Eraser
Matrix metalloproteinase 16	MMP16	P51512	CHEMBL2200	Protease
Matrix metalloproteinase 12	MMP12	P39900	CHEMBL4393	Protease
14-3-3 protein gamma	YWHAG	P61981	CHEMBL1293296	Unclassified protein
Hepatic lipase	LIPC	P11150	CHEMBL2127	Enzyme
Endothelial lipase	LIPG	Q9Y5X9	CHEMBL5080	Hydrolase
WD repeat-containing protein 5	WDR5	P61964	CHEMBL1075317	Unclassified protein
Serotonin 1a (5-HT1a) receptor	HTR1A	P08908	CHEMBL214	Family A G protein-coupled receptor
Matrix metalloproteinase 9	MMP9	P14780	CHEMBL321	Protease
Aminopeptidase N	ANPEP	P15144	CHEMBL1907	Protease
Glutaminyl-peptide cyclotransferase	QPCT	Q16769	CHEMBL4508	Enzyme
Carbonic anhydrase XIII	CA13	Q8N1Q1	CHEMBL3912	Lyase
Carbonic anhydrase VB	CA5B	Q9Y2D0	CHEMBL3969	Lyase
Matrix metalloproteinase 7	MMP7	P09237	CHEMBL4073	Protease
Glutamate receptor ionotropic kainate 2	GRIK2	Q13002	CHEMBL3683	Ligand-gated ion channel
CMP-N-acetylneuraminate-beta-1,4-galactoside alpha-2,3-sialyltransferase	ST3GAL3	Q11203	CHEMBL3596076	Transferase
Alpha-(1,3)-fucosyltransferase 7	FUT7	Q11130	CHEMBL3596077	Transferase
Fucosyltransferase 4	FUT4	P22083	CHEMBL4996	Enzyme

Coagulation factor VII/tissue factor	F3 F7	P13726 P08709	CHEMBL2095194	Protease
Metabotropic glutamate receptor 2	GRM2	Q14416	CHEMBL5137	Family C G protein-coupled receptor
Purinergic receptor P2Y12	P2RY12	Q9H244	CHEMBL2001	Family A G protein-coupled receptor
Gamma-secretase	PSEN2 PSENEN NCSTN APH1A PSEN1 APH1B	P49810 Q9NZ42 Q92542 Q96BI3 P49768 Q8WW43	CHEMBL2094135	Protease
Cyclooxygenase-2	PTGS2	P35354	CHEMBL230	Oxidoreductase
TNF-alpha	TNF	P01375	CHEMBL1825	Secreted protein
